# Pivotal Role of FBXW4 in Glioma Progression and Prognosis

**DOI:** 10.1155/2024/3005195

**Published:** 2024-09-30

**Authors:** Kun Chen, Lei Pu, Yuzuo Hui

**Affiliations:** ^1^ Department of Neurosurgery LiaoCheng People's Hospital, Liaocheng 252000, Shandong, China; ^2^ Department of Brain Disease Liaocheng Traditional Chinese Medicine Hospital, Liaocheng 252000, Shandong, China

## Abstract

**Backgrounds:**

Glioma stands as one of the most formidable brain tumor types, with patient outcomes remaining bleak even in the face of advancements in treatment modalities. FBXW4, a constituent of the F-box and WD repeat domain-containing protein family, is recognized for its participation in diverse cellular activities, including those related to tumor dynamics. Yet, the therapeutic relevance and specific role of FBXW4 in the context of glioma are not well defined. This study aims to elucidate the functional dynamics and significance of FBXW4 in glioma cases.

**Methods:**

This research undertook a comprehensive analysis of FBXW4's expression patterns and clinical relevance in glioma by harnessing data from the TCGA and GTEx databases.

**Results:**

The investigation revealed a distinct downregulation of FBXW4 in glioma tissues compared to normal brain counterparts, with a pronounced correlation between FBXW4 levels and disease severity. Intriguingly, FBXW4 expression inversely related to WHO tumor grades, with the most advanced grade IV gliomas exhibiting the lowest FBXW4 levels, whereas grade II tumors demonstrated the highest. Cases presenting with IDH1/2 mutations or 1p/19q codeletions were also associated with elevated FBXW4 levels. Furthermore, diminished FBXW4 expression aligned with an increased risk of mortality.

**Conclusions:**

The findings suggest that FBXW4 holds promise as a prognostic marker and a potential therapeutic avenue in glioma management. Nonetheless, future research is imperative to decode the intricate signaling pathways involving FBXW4 and to understand its broader clinical ramifications in glioma treatment paradigms.

## 1. Introduction

Glioma, a devastating form of primary brain tumor originating from glial cells, remains a formidable challenge in oncology [1]. These tumors constitute a significant proportion of all central nervous system neoplasms, making them a matter of paramount clinical concern [2]. Gliomas encompass a spectrum of malignancy grades, with glioblastoma multiforme (GBM), the most aggressive subtype, characterized by relentless growth and dismal prognosis [3]. The intricate interplay of genetic, molecular, and environmental factors underlies the pathogenesis of glioma, presenting a complex landscape for researchers and clinicians alike [4]. Despite advancements in therapeutic modalities, the treatment of gliomas continues to be limited by their infiltrative nature within the brain, making complete surgical resection challenging and recurrence common [5]. As such, elucidating the molecular mechanisms driving gliomagenesis and identifying novel therapeutic targets holds immense promise in advancing our understanding of this enigmatic disease and improving patient outcomes [6].

Recent investigations have underscored the pivotal role played by dysregulated components of the ubiquitin-proteasome system (UPS) in tumorigenesis [7]. F-box proteins, crucial regulators of protein degradation, have garnered attention in this context [8–10]. The FBXW (F-box and WD repeat domain containing) family, encompassing a diverse group of F-box proteins, has emerged as a critical player in the pathogenesis of various malignancies [11]. These F-box proteins serve as components of the Skp1-Cul1-F-box (SCF) ubiquitin ligase complex, regulating the ubiquitination and subsequent degradation of specific target proteins [12]. In the context of cancer, several FBXW family members have been implicated in tumorigenesis and tumor progression [13]. For instance, FBXW2 suppresses breast tumorigenesis by targeting AKT-Moesin-SKP2 axis [14]. Consistently, FBXW7, also known as CDC4, acts as a tumor suppressor in numerous cancers, including colorectal cancer [15] and acute myeloid leukemia [16], by targeting oncoproteins such as Cyclin E and c-Myc for degradation. Conversely, FBXW5 has been shown to promote the proliferation of breast cancer cells through the degradation of the tumor suppressor p53 [17]. Moreover, FBXW8 has been associated with the regulation of cell cycle progression and the ubiquitination of tumor suppressors in malignancies [18, 19]. These examples underscore the significance of FBXW family members in the intricate landscape of cancer biology, with each member exerting distinct roles in different malignancies.

The primary objective of this research is to conduct an exhaustive investigation into the multifaceted role of FBXW4 (F-box and WD repeat domain containing 4) in glioma. This study takes a comprehensive approach by harnessing the power of integrated analyses, merging extensive genomic datasets from the Cancer Genome Atlas (TCGA) and Genotype-Tissue Expression (GTEx). Through this amalgamation of clinical insights, we aim to unravel the clinical relevance and intricate molecular mechanisms underlying FBXW4's engagement in glioma. Ultimately, this research aspires to unveil new horizons for therapeutic interventions in the context of glioma.

## 2. Methods

### 2.1. Data Collection and Computational Analysis

For our research, we obtained RNA sequencing data in the Fragments Per Kilobase per Million (FPKM) format along with clinicopathological information from a total of 689 glioma samples and 1157 normal brain samples. These datasets were sourced from the Cancer Genome Atlas (TCGA) and the Genotype-Tissue Expression (GTEx) databases. We also extracted relevant clinicopathological data specific to glioma patients [20]. To discern any distinctions in clinicopathological parameters, we conducted comparisons between groups with high and low FBXW4 expression levels. Logistic regression analysis was employed to assess the relationship between FBXW4 expression and the clinicopathological characteristics of individuals with glioma.

### 2.2. Survival Analysis

To explore the survival outcomes of glioma patients, we utilized the survival data from the TCGA-glioma dataset. Kaplan–Meier survival analysis, as well as univariate and multivariate Cox regression analyses, was carried out. Progression-free survival refers to time from treatment to disease progression (or death); disease-free survival refers to time from treatment until the recurrence of disease (or death) after undergoing curative-intent treatment. These analyses were performed to evaluate the impact of FBXW4 expression alongside other clinicopathological factors on the survival prospects of glioma patients.

### 2.3. Statistical Analysis

All statistical analyses and data visualizations were performed using R (version 4.1.3). Statistical significance was defined as a threshold of *p* < 0.05. In our presentation of statistical results, asterisks were employed to denote significance levels: ^∗^ for *p* < 0.05, ^∗∗^ for *p* < 0.01, and ^∗∗∗^ for *p* < 0.001.

### 2.4. Ethical Considerations

The Ethics Committee of Liaocheng People's Hospital has determined that no ethical approval is required for this study, as it involves a public-database analysis. An exemption from obtaining informed consent has been granted by the Ethics Committee for this specific study.

## 3. Results

### 3.1. FBXW4 Expression and Demographic Characteristics

As shown in Table 1, in the cohort of 699 glioma patients, FBXW4 expression levels were examined and classified into low (*n* = 349) and high (*n* = 350) categories. Analysis of demographic characteristics revealed no significant difference in gender distribution between the low and high FBXW4 expression groups (female: 21% vs. 21.6% and male: 28.9% vs. 28.5%; *p*=0.785). Similarly, racial composition between the two groups showed no substantial variation (Asian/Black/African American: 3.9% vs. 2.8% and White: 46.1% vs. 47.2%; *p*=0.222). However, a significant difference was observed in the age distribution; patients aged ≤60 years were more prevalent in the high FBXW4 expression group (45.5%) compared to the low expression group (34%), while the opposite trend was seen for patients aged >60 years (4.6% in the high vs. 15.9% in the low expression group; *p* < 0.001).

### 3.2. FBXW4 Expression and Clinical Characteristics

The analysis further extended to clinical characteristics, where FBXW4 expression showed significant associations. The World Health Organization (WHO) grade of tumors varied distinctly between the two groups, with a higher prevalence of Grade 2 tumors in the high FBXW4 expression group (25.4% vs. 9.7%) and a predominance of Grade 4 tumors in the low expression group (25.6% vs. 0.8%; *p* < 0.001). Histological types also demonstrated a significant association with FBXW4 expression. Astrocytomas (11.7% vs. 16.3%), oligoastrocytomas (6% vs. 13.3%), oligodendrogliomas (8.9% vs. 19.7%), and glioblastomas (23.3% vs. 0.7%) were all differentially represented between low and high FBXW4 expression groups (*p* < 0.001). Moreover, the isocitrate dehydrogenase (IDH) status was significantly correlated with FBXW4 expression. The high expression group had a substantially higher proportion of IDH-mutated tumors (46.6% vs. 17.7%), while the low expression group had more IDH1/2 wild-type tumors (31.8% vs. 3.9%; *p* < 0.001). Similarly, the presence of 1p/19q codeletion was markedly different between the groups, with the high FBXW4 expression group showing a higher frequency of codeletion (19.7% vs. 5.2%; *p* < 0.001).

**Figure 1:**
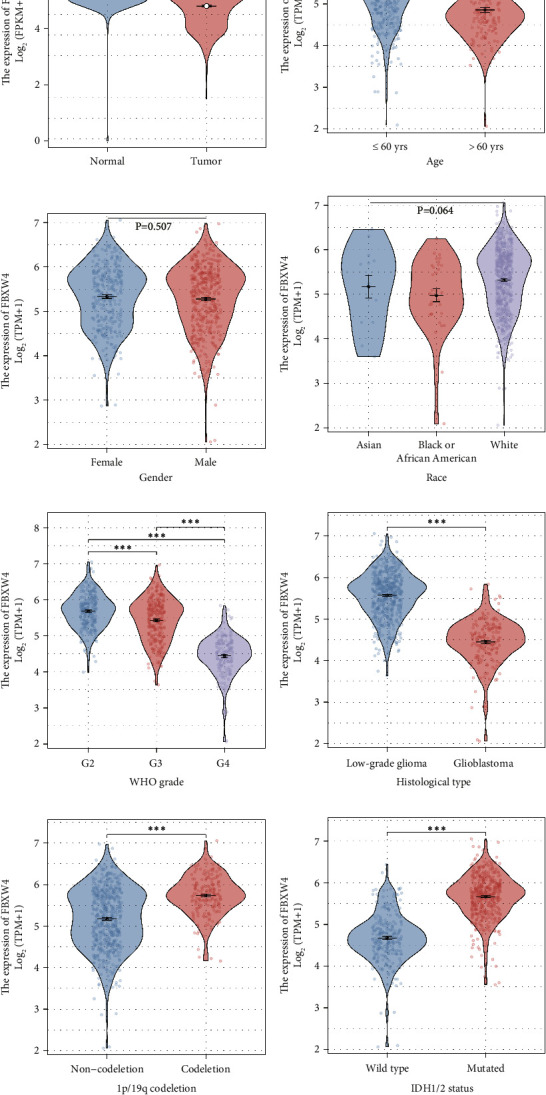
The correlation between FBXW4 expression and disease status in glioma. (a) RNAseq data from GTEx and TCGA datasets revealed that glioma tissues had significantly lower FBXW4 expression levels than normal brain tissues (*p* < 0.001). (b) Patients with elder age showed lower FBXW4 level (*p* < 0.001). (c) FBXW4 showed no statistically significant difference between males and females (*p*=0.507). (d) FBXW4 showed no statistically significant difference among different racial (*p*=0.064). (e) FBXW4 expression was negatively correlated with the WHO grade, with grade IV samples exhibiting the lowest FBXW4 levels and grade II samples showing the highest levels (*p* < 0.001). (f) Glioblastoma patients showed significantly lower FBXW4 level than those with low-grade glioma types (*p* < 0.001). (g) Patients with 1p/19q codeletion exhibited higher FBXW4 levels (*p* < 0.001). (h) Patients with IDH1/2 mutation showed higher FBXW4 levels (*p* < 0.001).

Consistent data were observed in Figure 1. In brief, analysis of RNAseq data from GTEx and TCGA revealed that glioma tissues express FBXW4 at significantly lower levels compared to normal brain tissues, with older patients typically presenting reduced levels of FBXW4. Gender and racial background did not significantly affect FBXW4 expression. Interestingly, FBXW4 levels were inversely related to the WHO grade of the tumor, with the lowest levels observed in the most severe grade IV gliomas. Similarly, patients with glioblastoma exhibited notably lower FBXW4 levels compared to those with lower-grade glioma. In contrast, genetic markers like 1p/19q codeletion and IDH1/2 mutation were associated with higher FBXW4 expression, highlighting a complex interplay between FBXW4 levels and glioma characteristics.

The results underscore a significant correlation between FBXW4 expression levels and various demographic and clinical characteristics in glioma patients. Notably, the expression level of FBXW4 is significantly associated with age, the WHO grade, histological type, IDH1/2 status, and 1p/19q codeletion status, suggesting that FBXW4 could potentially serve as a biomarker for glioma characterization and prognosis.

### 3.3. FBXW4 is a Prognostic Biomarker of Glioma

**Figure 2:**
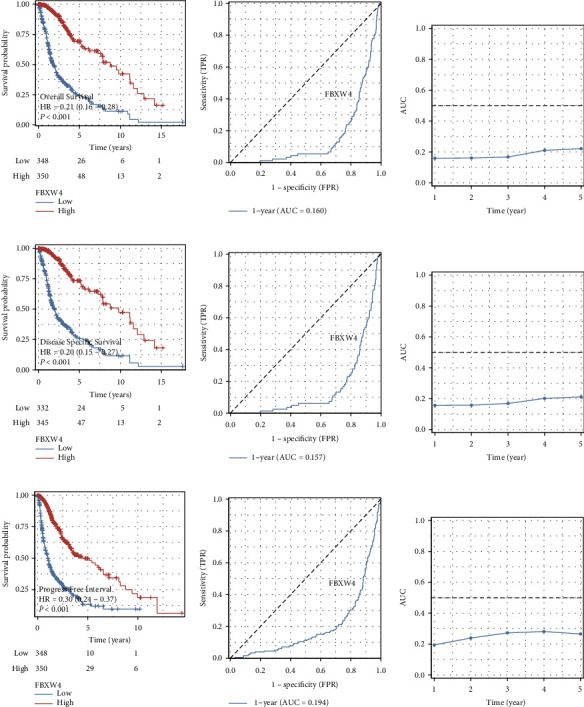
Prognostic significance of FBXW4 in glioma. (a–c) Kaplan–Meier survival analysis to explore the different overall survival patterns of glioma patients with low- or high-FBXW4 level (a), which is supplemented with time-dependent ROC curve (b) and AUC curve (c). (d–f) Kaplan–Meier survival analysis to explore the different disease-specific survival patterns of glioma patients with low- or high-FBXW4 level. (g–i) Kaplan–Meier survival analysis to explore the different progress-free survival patterns of glioma patients with low- or high-FBXW4 level.

In the univariate analysis encompassing 698 glioma patients (Table 2), age emerged as a prominent risk factor, with individuals over 60 displaying a markedly increased hazard ratio (HR = 4.696, *p* < 0.001). While gender differences suggested a marginally elevated risk for males, this trend did not achieve statistical significance. Racial disparities were examined, though they did not significantly influence survival outcomes. Notably, a stark distinction in survival rates was evident across the WHO tumor grades, with higher grades correlating with a substantial surge in risk, particularly pronounced in G4 tumors (HR = 18.600, *p* < 0.001). The molecular landscape also held prognostic value; IDH1/2 mutations and 1p/19q codeletion were associated with markedly improved survival prospects. Furthermore, high FBXW4 expression emerged as a significant protective factor, markedly reducing the risk of poor outcomes (Figures 2(a), 2(b), 2(c), 2(d), 2(e), 2(f), 2(g), 2(h), and 2(i)), underscoring its potential as a biomarker for glioma prognosis.

The multivariate analysis refined these insights, accounting for the interplay between various factors (Table 2). Age retained its significance as a risk factor for diminished survival, albeit with a moderated hazard ratio (HR = 1.518, *p*=0.008). The relative risk associated with gender and race was reassessed, with neither reaching statistical significance, suggesting a nuanced interrelation with other variables. The stratification by the WHO tumor grades confirmed their predictive value, particularly highlighting the escalated risk associated with G4 tumors (HR = 3.516, *p* < 0.001). In this comprehensive model, the protective influence of IDH1/2 mutations remained robust (HR = 0.317, *p* < 0.001). The role of FBXW4 expression was reaffirmed, with high levels continuing to denote a significantly lower risk (HR = 0.651, *p*=0.040), asserting its relevance in the context of multifactorial influences on glioma patient survival.

These univariate and multivariate findings highlighted the significance of each factor in isolation and in conjunction with others, with an emphasis on the prognostic implications of FBXW4 expression levels.

## 4. Discussions

In this study, we harness large-scale genomic datasets from TCGA to meticulously evaluate FBXW4's clinical relevance in glioma patients and juxtapose it with expression patterns in normal brain tissue obtained from GTEx. Interestingly, the role of FBXW4 seems distinct in different tumor types. For example, FBXW4 is highly expressed and associated with unfavorable prognosis in acute myeloid leukemia [21] while showed no prognostic relevance in lung cancer [22]. Transient or stable depletion of FBXW4 activates the BCL-2 survival pathway [23]. Several other aspects regarding its function had also been reported such as serving as the FBXW4 fusion partner of TGFBR3 in pleomorphic hyalinizing angiectatic tumor and circFBXW4 regulates human trophoblast cell proliferation and invasion [24, 25]. Moreover, FBXW4 acts as a protector of FOLFOX-based chemotherapy in metastatic colorectal cancer identified by coexpression network analysis [26]. In contrast with the potential oncogenic role in myeloid leukemia, expression of FBXW4 was decreased in breast cancer and may serve as a tumor suppressor [27]. Consistently, the inhibition of glioma cell proliferation by FBXW4 in our study opens up new avenues for therapeutic interventions. The ability to modulate cell growth through targeted manipulation of FBXW4 expression levels could offer a strategic pivot point in glioma treatment, moving away from traditional, more invasive treatment modalities [28]. Furthermore, the specificity with which FBXW4 impacts cell proliferation points towards its potential role in the delicate balance of cellular mechanisms, possibly influencing pathways like cell cycle regulation, apoptosis, or DNA repair mechanisms, commonly dysregulated in cancer pathogenesis.

However, while the results are promising, it is crucial to approach them with a degree of caution. The complexity of glioma as a disease—marked by heterogeneity in genetic, molecular, and histological profiles—necessitates a comprehensive understanding of FBXW4's role in the broader context of these variables [20]. The interplay between FBXW4 and other molecular pathways within glioma cells remains a territory ripe for exploration. For instance, future studies could focus on delineating the downstream targets of FBXW4, unraveling the mechanistic pathways through which it exerts its tumor-suppressive effects, and identifying potential resistance mechanisms that might limit the efficacy of FBXW4-based therapies.

Current glioma therapies often fall short due to the tumor's invasive nature and complex genetic variability, making complete surgical resection difficult and recurrence likely. FBXW4's strong correlation with glioma severity and patient outcomes makes it an excellent biomarker for selecting participants in clinical trials, allowing for the identification of individuals who might gain the most from novel targeted therapies. Furthermore, integrating FBXW4 inhibitors with treatments aimed at other essential glioma pathways could provide a more comprehensive approach to combating the disease's complexity and resistance to current treatments. For example, therapies that target both FBXW4 and the PI3K/AKT/mTOR pathway, commonly active in these tumors, could enhance overall effectiveness and reduce the likelihood of recurrence. The distinctive downregulation of FBXW4 in aggressive gliomas and its impact on prognosis underline its potential as a focal point for new therapeutic developments. Such strategies could not only improve therapeutic outcomes by addressing tumor progression through multiple biological mechanisms but also spearhead advances in personalized medicine and deepen our understanding of cancer biology.

Despite the promising insights gleaned from this study, it is important to acknowledge its other inherent limitations, which must be addressed to fully understand the implications of our findings. The behavior of glioma cells in the controlled environment of a laboratory may differ significantly from their behavior within the complex biological systems of the human body, where interactions with the immune system, blood-brain barrier, and other factors play pivotal roles. Therefore, further in vivo study as well as in vitro experiments should be conducted for validation. In addition, here we did not provide a complete picture of the signaling pathways and molecular interactions involved downstream of FBXW4. A deeper understanding of these pathways is crucial for translating these findings into therapeutic strategies. Addressing these limitations in future research, through the incorporation of in vivo/vitro studies, a broader array of glioma subtypes, and comprehensive molecular analyses, will be paramount in validating and extending the applicability of our findings to glioma treatment and management.

## 5. Conclusions

In conclusion, our study adds a significant piece to the puzzle of glioma pathogenesis and therapy. By highlighting the tumor-suppressive role of FBXW4, it lays the groundwork for future research aimed at harnessing its potential for therapeutic benefit. While the road ahead is long and complex, the insights gained from this study illuminate promising pathways for innovative, targeted approaches to glioma treatment. As we advance, it is imperative that these findings are meticulously validated and contextualized within the intricate tapestry of glioma biology, ensuring that the leap from laboratory discovery to clinical application is both scientifically sound and ethically responsible.

## Figures and Tables

**Table 1:** Correlations between FBXW4 level and glioma patients' characteristics.

Characteristics	Low expression of FBXW4	High expression of FBXW4	*p* value
*n*	349	350	
Age, *n* (%)			<0.001
≤60 yrs	238 (34%)	318 (45.5%)	
>60 yrs	111 (15.9%)	32 (4.6%)	
Gender, *n* (%)			0.785
Female	147 (21%)	151 (21.6%)	
Male	202 (28.9%)	199 (28.5%)	
Race, *n* (%)			0.222
Asian/Black/African American	27 (3.9%)	19 (2.8%)	
White	316 (46.1%)	324 (47.2%)	
WHO grade, *n* (%)			<0.001
G2	62 (9.7%)	162 (25.4%)	
G3	108 (17%)	137 (21.5%)	
G4	163 (25.6%)	5 (0.8%)	
Histological type, *n* (%)			<0.001
Astrocytoma	82 (11.7%)	114 (16.3%)	
Oligoastrocytoma	42 (6%)	93 (13.3%)	
Oligodendroglioma	62 (8.9%)	138 (19.7%)	
Glioblastoma	163 (23.3%)	5 (0.7%)	
IDH1/2 status, *n* (%)			<0.001
Wild type	219 (31.8%)	27 (3.9%)	
Mutated	122 (17.7%)	321 (46.6%)	
1p/19q codeletion, *n* (%)			<0.001
Noncodeletion	306 (44.2%)	214 (30.9%)	
Codeletion	36 (5.2%)	136 (19.7%)	

**Table 2:** Survival analyses.

Characteristics	Total (*N*)	Univariate analysis		Multivariate analysis	
		Hazard ratio (95% CI)	*p* value	Hazard ratio (95% CI)	*p* value
Age	698				
≤60 yrs	555	Reference		Reference	
>60 yrs	143	4.696 (3.620–6.093)	<0.001	1.518 (1.113–2.070)	0.008
Gender	698				
Female	297	Reference		Reference	
Male	401	1.250 (0.979–1.595)	0.073	1.208 (0.919–1.588)	0.175
Race	685				
Asian/Black/African American	46	Reference			
White	639	0.817 (0.499–1.337)	0.421		
WHO grade	636				
G2	223	Reference		Reference	
G3	245	2.967 (1.986–4.433)	<0.001	1.807 (1.170–2.792)	0.008
G4	168	18.600 (12.448–27.794)	<0.001	3.516 (1.862–6.640)	<0.001
Histological type	698				
Astrocytoma and oligodendroglioma	395	Reference		Reference	
Glioblastoma and oligoastrocytoma	303	3.132 (2.445–4.012)	<0.001	1.224 (0.780–1.921)	0.380
IDH1/2 status	688				
Wild type	246	Reference		Reference	
Mutated	442	0.116 (0.089–0.151)	<0.001	0.317 (0.206–0.487)	<0.001
1p/19q codeletion	691				
Noncodeletion	520	Reference		Reference	
Codeletion	171	0.225 (0.147–0.346)	<0.001	0.765 (0.456–1.282)	0.309
FBXW4 level	698				
Low	348	Reference		Reference	
High	350	0.208 (0.157–0.276)	<0.001	0.651 (0.433–0.981)	0.040

## Data Availability

The data used to support the findings of this study are available from the corresponding author upon reasonable request.
